# West Texas Medical Association

**Published:** 1902-12

**Authors:** W. B. Russ

**Affiliations:** Secretary


### Society Notes.

### West Texas Medical Association.

The November meeting of the West Texas Medical Association was held on Thursday night, the 20th, the newly elected President, T. T. Jackson, in the chair.

Dr. T. J. Turpin, of Monterey, Mexico, and Dr. R. H. Harrison, Jr., of Columbus, Texas, were elected to membership, bringing the total number of members to eighty-three, an increase of thirty-three in less than twelve months.

On motion by Dr. Robt. E. Moss, seconded by Drs. W. M. Wolff and W. E. Luter, a committee was appointed to arrange programs for a series of quarterly "social sessions" which are to be held during the coming year. It is intended that these sessions shall constitute an important feature in the future.

Dr. F. Paschal, Chairman of the Committee on Arrangements, called attention to the fact that the State Medical Association will hold its 1903 meeting in this city in April. He hopes that all the members of the local society will assist in entertaining the large number visitors who will be present at that time.

On motion by Dr. W. B. Russ, seconded by Colonel P. A. J. Cleary, the President was authorized to appoint a committee to formulate a compromise draft of the constitution and by-laws of the State Association, to be offered next April as a substitute for the so-called "majority" and "minority" drafts presented at the 1902 meeting of that society. Dr. Russ was appointed chairman of this committee and the other members are to be announced later. The West Texas Medical Association is anxious to be the means of bringing about an amicable settlement of the difficulties that arose during the discussion of this question at Dallas.

Before the close of the business session Dr. Thos. P. Lloyd was unanimously elected Treasurer to serve until October, 1903.

Dr. Sigmund Burg read an interesting essay on "The Management of Occipito-posterior Positions." This paper was discussed by Drs. B. F. Kingsley, J. S. Lankford and F. Paschal.

Major Chas. F. Mason, U. S. A., reported two unusual and extremely interesting cases of appendicitis and presented for examination the specimens removed at the operations.

The members present were: Colonel Peter A. J. Cleary, U. S. A.; Major Chas. F. Mason, U. S. A.; Drs. S. Burg, A. A. Brown, P. Baldesarelli, F. Paschal, Clarence Warfield, Robt. E. Moss, T. P. Lloyd, G. G. Watts, A. Garwood of New Braunfels, R. Robinson, B. F. Kingsley, W. E. Luter, J. S. Lankford, Jas. Bartlett, E. C. Calvin, T. T. Jackson, W. M. Wolff and W. B. Russ.

W. B. Russ,
Secretary.

